# Pathology

**Published:** 1858-07

**Authors:** 


### PATHOLOGY.

### V. *On Spontaneous Primary Pyæmia.* By PROF. C. A. WUNDERLICH.

THE author communicates five cases of spontaneous primary pyæmia. It is well known that the existence of this disease is doubted by many pathologists, as Castelnau, Ducrest, and others. In consideration of the confusion of language and ideas as yet existing in regard to the pathology of the disease, Prof. Wunderlich thought it necessary to preface his report by some preliminary remarks, in order to make himself better understood.

Pathologists have long since ceased to understand by the term "pyæmia" a constant and demonstrable morbid condition of the blood; nor is its application at present confined merely to such cases of the disease in which the product of morbid action is pus. The term has acquired a conventional signification which is rather contradictory to the etymology of the word, as well as to the idea originally attached to it. Pathologists have accustomed themselves to use the term as designating that morbid condition of the system in which, without specific cause, (as syphilitic, variolous, carcinomatous infection, &c.,) certain acute morbid processes, inflammatory in their character, or connected with inflammation, (as extravasation, formation of ichor, &c.,) are developed in several organs or tissues at the same time; the disease of the more or less numerous and distant parts thus attacked not being traceable to any local, exciting cause.

The absence of pus in the blood, the non-existence of any palpable morbid change in it is therefore no objection to the diagnosis of pyæmia. We do not know the essential morbid processes by which the multiplied local affections are brought about; but as they appear in many places at the same time, and without local cause, we have to assume a constitutional disturbance, which pathologists have termed "pyæmia."

In regard to the terms "spontaneous" and "primary," it is unquestionable that, as in nature generally, so also in pathological processes, nothing can happen *spontaneously*. The term means simply that the cause of disease is unknown, and particularly that no infection from external sources has taken place. We are just as far from maintaining that the morbid changes of a pyæmic character observed in the different cases have been *primary* disturbances. What we wish to express by the term is, that these changes were neither occasioned by any local suppuration nor consequent upon any severe general disease, as typhus, scurvy, &c., constitutional disorders of the system in which, as it is well known, the sudden occurrence of suppuration and formation of ichor in different parts of the body is frequently observed.

The following are the cases reported by Prof. Wunderlich:—

CASE I.—A strong and healthy-looking coachman, twenty-five years of age, fell suddenly, and injured his right trochanter. He complained of pain, and the parts were bruised; there was, however, no inflammation developed in the spot, as was confirmed afterwards by post-mortem examination. Symptoms of an apparently typhoid fever soon set in, with very high temperature, delirium, drowsiness, tumefaction of the spleen, petechiæ, albuminuria, but no chills. Death took place on the fifteenth day. The autopsy revealed multiple yellow infiltration of the spleen and left kidney; catarrhal inflammation of the pelvis of the latter, and of the intestines; extravasations underneath the endocardium, and in the vastus externus muscle of the right side.

CASE II.—A cabinet-maker, thirty-nine years of age, occupying a damp dwelling, fell from a ladder a few steps, and struck his head slightly. He felt very weak immediately afterwards; had pain in his fore-arms and feet, without having injured them. Severe symptoms soon set in, partly cerebral, partly pulmonic, partly abdominal, (diarrhœa, painful abdomen, enlargement of spleen;) albuminuria, with admixture of blood; violent fever without chills; temperature 32·5° R.; very frequent pulse, and extravasation underneath the skin. Death on the twelfth day.

Post-mortem appearances.—Double suppurative pleuritis; two abscesses in the left, and numerous lobular infiltrations in the right lung; enlargement and softening of spleen without deposits; both kidneys contained numerous, disseminated, purulent deposits; croupy inflammation of the pelvis of the right kidney and ureters; intestines normal; a completely isolated collection of pus in the sheath of the tendon of the left extensor carpi radialis muscle; bones and vessels unaffected.

CASE III.—A man of rather slender form, twenty-three years of age, living in good circumstances, was taken ill with severe diarrhœa, after having slept near a damp wall in a badly built summer-residence. On the evening of the same day he had a chill of fifteen minutes duration, which recurred after two days. Soon afterward supervened pain and swelling of the joints, delirium, high temperature, icterus, rapid collapse. Death on the twelfth day. Body decomposing with remarkable rapidity; biliary apparatus normal; spleen double its natural size, and softened; Peyer's glands in the ileum of dark-gray color; enlargement of a number of the solitary follicles; mesenteric glands somewhat enlarged; the right knee-joint contained thick, dark-yellow pus, and its capsule was slightly reddened in some places; the bones and periosteum normal. In the rectus femoris muscle there were several yellow semi-fluid deposits, and disseminated abscesses of the size of a filbert, containing dark-yellow pus.

Case IV.—A servant girl, twenty-six years of age, got thoroughly wet while washing. The day previous to it her catamenial discharge had commenced. On rising in the morning she was taken with dizziness, syncope, and afterwards a chill, which lasted almost the whole day. The next day she had very intense fever, pectoral symptoms, pain in several joints, albuminuria, liquid stools, an eruption similar to ecthyma, and blisters on her hands and feet. Death on the fourth day. On post-mortem examination, numerous extravasations and sanguineo-purulent deposits were found in the cutis; purulent myocarditis, hypendo- and hypocarditis, with partial sugillations; pleuritis; œdema of the lung; many small abscesses in the thyroid gland, liver, and kidneys; spleen softened and enlarged; recent extravasation in the brain.

Case V.—A healthy man, twenty-one years of age, employed in a factory, was taken ill, without any assignable cause, with the symptoms of severe typhus. On the seventh day (commencement of our observation) the unusually high temperature for typhus (32·9° R.) was first noticed. On the ninth day the joints became affected, and a roseolar eruption broke out. Albumen in the urine, which had been noticed, now disappeared. The skin, discolored by icterus, was covered by pemphigus, blisters, and numerous ecthymatous pustules. Death took place on the fifteenth day. On an autopsy, abscesses were found in the areolar tissue of the neck, in the sterno-clavicular articulation, in the pectoral muscles, in the shoulder, and in the elbow-joint; bronchitis; on the right side limited infarction of the lung; slight pleuritis; in the stomach ecchymoses; some glands of Peyer prominent; slight swelling of the mesenteric glands; spleen enlarged, softened; catarrhal inflammation of kidneys; soft coagulum adhering to the inner wall of the right iliac vein.

Prof. Wunderlich sums up his observations as follows:—

1. There occur certain cases of disease which can only be regarded as cases of idiopathic pyæmia. All the anatomical characters of pyæmia are present. Numerous foci of morbid action, bearing the characters of inflammation, its preliminary states, and its products, are spread over different tissues and organs; but there is no evidence of causes known to produce pyæmia, such as injuries with consequent local suppuration, or the puerperal state, phlebitis or endocarditis, formation of ichor in different localities, introduction of putrefying substances into the system by dissecting wounds, or infection from miasmatic agents; nor is it apparent, that a specific process, communicated by contagion (such as variola, glanders, &c.,) or tuberculosis, co-operate in giving rise to these numerous inflammations. The disorder of the constitution is, in such cases, as far as we can judge from our present state of knowledge, of a completely self-generating character.

2. The constitutional disease, appearing in the form of idiopathic pyæmia, can be developed in individuals enjoying complete health. In all five cases the patients had not suffered from any disturbance of health, or only to so slight a degree as that it could not have any connection with the grave disease by which they were attacked. The essential causes are quite unknown. Predisposing causes are just as little apparent. In three cases, however, exposure to moisture (sleeping in a damp room in two, getting wet in one) seems to have produced the disease, or determined its outbreak. The disease commenced in January in one case; in February in three, in July (moist and cold summer) in one.

3. The anatomical changes found distributed over a great number of parts of the body were, in our five cases, the following:—Plastic infiltration of the tissues, or plastic exudations on serous membranes in all five cases; catarrhal or croupy affections of the mucous membranes in all cases; softening and enlargement of the spleen in all cases: suppuration in four cases; extravasation of blood in three cases; hemorrhagic exudations in two cases; œdema in two cases; early putrefaction of the body in two cases; soft, abnormally colored coagulum in a vein (which, however, did not correspond to the affected periphery) in one case.

4. The number of affected organs was very great. No circumstance served to explain the reason why some of these were attacked in one case and escaped in the other. The following changes attracted notice:—In the brain, large extravasation in one case; in the lips, abscess in one; in the areolar tissue of the neck, suppuration in one; in the thyroid gland, abscess in one; in the heart, infiltration and abscess in one, and extravasation in two; in the bronchial tubes, catarrhal inflammation in two; in the lungs, œdema in one, infiltration and abscess in one; bloody infarction in one; in the pleura, plastic exudations in three, purulent and hemorrhagic exudation in one; in the stomach, extravasations in one; in the liver, small abscesses in one; in the spleen, softening and enlargement in each case, together with numerous infiltrations in one; in the kidneys, numerous infiltrations or abscesses in three; in the pelvis of the kidneys, catarrh in two, and croupy exudation in it and the ureter in one; in the areolar tissue, suppuration in one, œdema in one; in the muscles, infiltrations and abscesses in two, extravasation in one; in the sheath of tendons, abscess in one; in the joints, abscesses in two; in the skin, extravasations in four, purulent and sanguineo-purulent pustules and blisters in two, icterus in two, erysipelas in one.

5. The first decided symptom of disease was, in three cases, syncope, in consequence of which the patients fell down. The injuries thus received were slight, and could only in the beginning be considered of importance; the further course, as well as the anatomical investigation of the injured parts, proved that these injuries could have exercised no influence upon the development of pyæmia. In three cases a chill was noticed among the first symptoms. In all cases a deep, general feeling of illness was present from the beginning.

6. The general type of the disease, and the combination of the different symptoms was of a kind that, in all the cases at their beginning, and in several until death, the diagnosis could not be determined.

In three cases (I., II., and V.) the characters and combinations of symptoms resembled, more or less, typhus; in two of them (I. and V.) affections of the follicles in the lower part of the small intestines were present, though not in a degree which could justify the diagnosis of typhus on anatomical grounds; in case II. no follicular catarrh was found, while its existence in case III. was not accompanied with typhoid symptoms. In case II. the diagnosis wavered in the beginning between typhus and acute tuberculosis. The commencement of case III. was of the character of intermittent fever. Cases III. and V. could have been considered rheumatic arthritis or malignant icterus. Cases I. and II. resembled local acute disease of the kidneys.

7. The particular symptoms were exceedingly various; a number of them, however, were constant in all cases, no matter what form the local changes of the organs had assumed.

The chill was repeated in only one case. The other symptoms of fever were in all cases of great intensity. The temperature rose and remained for several days at a height which, in less dangerous diseases, hardly occurs in a transitory manner (32·5–33·0° R.;) it made only insignificant remissions in the morning, or none at all, a feature different from that in enteric typhus, and rose in one case, immediately before death, to the extremest point ever observed (34·2° R.)

A decided intermission, as observed in intermittent fever of even the worst kind, never took place. Thirst was in all cases very intense. The pulse corresponded generally to the temperature; it was found to be from 112 to 148 beats a minute, and diminished in frequency only for short intervals. A dicrotic pulse was observed several times.

The expression and feeling of prostration was very marked in all cases. All the patients sunk rapidly, and presented very soon, either temporarily or permanently, symptoms of collapse. Grave cerebral symptoms were present in all cases. Dizziness, headache, tinnitus aurium were complained of by the majority of patients; delirium was not absent in any case. The cerebral symptoms were not explained by anatomical lesions, and just in the case in which apoplexy finally occurred, (IV.) consciousness had been retained most perfectly during the course of the disease. Dyspnœa was considerable in four cases, and was in three in no proportion to the rather insignificant lesions in the respiratory organs. Complete loss of appetite, dryness or thick coating of the tongue were present from the beginning in all cases. Diarrhœa was observed in all cases; in most of them it occurred early, was at one time slight, at other times profuse. The stools were of more or less yellowish color, and did not show the stratification of typhoid discharges. Tumid, more or less tense abdomen was present in four cases, and in the same patients sensitiveness and gurgling in the ileo-cæcal region were noticed. Albuminuria was observed in four cases; in one it disappeared before death; bloody urine was discharged in two cases; pain in the joints was present in every case, even in those in which no anatomical lesions could be found in the joints or their vicinity. Besides the intensity of thirst, this was the principal complaint of the patients. The skin presented four times a cyanotic, twice an icteric discoloration. Numerous sudamina appeared in cases II. and V. A slight roseolar eruption was observed on the trunk in cases I., II., and V., petechiæ in the same, larger livid spots and sugillations in the same, and in case IV. In this case also an eruption of ecthymatous pustules, and blisters filled with blood and ichor, appeared on hands and feet; in case V. an eruption of ecthymatous pustules and pemphigus on the back.

8. The course of the disease was rapid in all cases, and the termination fatal.—(*Schmidt's Jahrb.*, 1858, No. 2, from *Arch. f. Physiol. Heilk.*, p. 89, 1857.)

